# Where to Live

**Published:** 1903-05-23

**Authors:** 


### EDITOR'S LETTER-BOX.

[Our Correspondents are reminded that prolixity is a great bar to publication, and that brevity of style and conciseness of statement greatly facilitate early insertion.]

### WHERE TO LIVE.

A LEADING ACTOR writes: "As a reader of your paper, might I ask if you could furnish me with the following information:—The most bracing and healthy localities to live in within 20 miles of London, specially with regard to delicate children?"

[Of London itself there is no healthier part than Hampstead, but if this be not far enough away, we should suggest either the Harrow and Stanmore district, or else the eastern part of Surrey south of Croydon. With regard to the former district, it is very bracing, and children do especially well there, but it has one drawback—rheumatic affections are common, perhaps on account of the clay soil. Generally speaking a gravel soil is the best, and the higher a house is above the sea-level the better.]

